# Application of circulating tumor cells scope technique on circulating tumor cell research

**DOI:** 10.1186/2052-8426-2-8

**Published:** 2014-03-10

**Authors:** Dawei Yang, Lijie Wang, Xiaochen Tian

**Affiliations:** Department of Pulmonary Medicine, Zhongshan Hospital, Fudan University, No. 180 Fenglin Rd, Shanghai, 200032 China; Department of Health Service Management, Second Military Medical University, No. 800 Xiangyin Rd, Shanghai, 200433 China; China Key Laboratory of Medical Molecular Virology, Shanghai Medical College, Fudan University, No. 138 Yixueyuan Rd, Shanghai, 200032 China

**Keywords:** CTCs, ISH, qRT-PCR, CTCscope

## Abstract

Circulating tumor cells (CTCs) are becoming promising biomarkers in several cancers, such as colon, prostate, and breast carcinomas. Independent research groups have reported a correlation between CTC numbers and patient prognosis. Even more, the development of personalized medicine gives physicians impetus to utilize the advancement of molecular characterization of CTCs. This review introduces a new technique, CTCscope, and compares it with the current methods of CTCs detection, with particular emphasis on cancer research, and discusses the future application of this new method from bench to bed-side.

## Review

### Introduction

Circulating tumor cells (CTCs) are becoming promising biomarkers in several cancers, such as colon, prostate, and breast carcinomas. Due to the lost expression of common epithelial markers by certain types of CTCs, such as EpCAM or the cytokeratin (CK), current positive selection strategies are usually with low sensitivity and efficiency [1, 2]. For example, most breast tumour stem cells are estrogen receptor negative, which could not be detected by positive selection method [3] (Table 1).

**Table 1:** **Comparison of current CTC enrichment and detection method with CTCscope technique**

Method	Enrichment	Detection	Sensitivity	Specificity	Sample volume	Cell morphology	Cell viability	Limitations	Advantages	Reference
CTCscope	Density of mononuclear cells (Ficoll centrifugation)	RNA ISH (Multiplex CTC specific mRNA)	High	High	Less blood sample	Good	Live cells	Not easy to perform in a clinical lab	Simple technique; EpCAM-negative cells can be isolated	Payne et al. 2012 [4]
CellSearch	Immunomagnetic enrichment of EpCAM-positive cells	IHC (CKs, CD45, DAPI)	Moderate; low in EpCAM negative cases	High	At least 7.5 mL blood sample	Poor	Live or dead cells	Cannot identify EpCAM-negative CTCs (such as tumour stem cells with estrogen receptor negative phenotype in breast cancers); expensive;	Easy to use semiautomated system; reproducibility; only assay approved by FDA	Peeters et al. 2013 [5]
AdnaTest	Detected only viable CK19-releasing	RNA isolation and multiplex PCR for tumour-specific transcripts (MUC1+/HER2+/EpCAM+)	High	High	5mL	Good	-	MUC1 is also expressed on activated T lymphocytes; Semiquantitative PCR	Enables the additional analysis of transcripts	Tewes et al. 2009 [6]
MagSweeper	Magnetic isolation	None	High	High	At least 7.5 mL blood sample	Good	Live cells	Low efficiency	No impact on the transcriptional profile of single cancer cell isolated	Talasaz et al. 2009 [7]
Cytometric analysis	Immunoflurorescent detection of antigen expression	None	Low	-	-	Good	Live cells	Dependent on expression of epithelial or tumor markers	Further characterization (FACS); multiple antibodies; morphology evaluation	Lu et al. 2010 [8]

**Figure 1:**
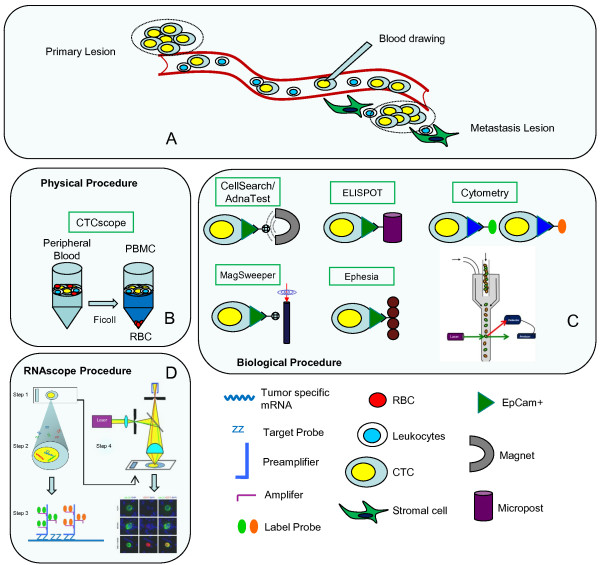
**Enrichment of CTCs from peripheral blood of cancer patients by physical or biological properties.** **A**: Transition of CTC from primary lesion to metastasis lesion. **B**: Physical properties include density (Ficoll centrifugation) **-** ***CTCscope***. **C**: Biological properties are based on the following: the expression of cell surface markers, including an epithelial cell adhesion molecule (EpCAM) for positive selection ***– CellSearch/AdnaTest***; anti-EpCAM antibodies conjugated with magnetic beads, for enriching CTCs in a magnetic field – ***MagSweeper***; anti-EpCAM antibodies on microposts or columns of nanobeads ***– ELISPOT/Ephesia***; anti-EpCAM antibodies conjugated to 3-μm beads to increase the size of CTCs before filtration ***- Cytometry***. **D**: Schematic of the RNAscope assay procedure ***- CTCscope***. In step 1, cells are fixed and permeabilized to allow for target probe access. In step 2, target RNA-specific oligonucleotide probes (Z) are hybridized in pairs (ZZ) to multiple RNA targets. In step 3, multiple signal amplification molecules are hybridized, each recognizing a specific target probe, and each unique label probe is conjugated to a different fluorophore or enzyme. In step 4, signals are detected using an epifluorescent microscope (for fluorescent label) (CTCscope image are reproduced from Payne et al. [4]) PBMC: peripheral blood mononuclear cells; CTC: circulating tumor cells; RBC: red blood cell.

In 2011, RNAscope or CTCscope was developed for detecting multiple tumour-specific marker mRNAs in tumor tissues [9, 10], as well as CTCs from blood [11]. In brief, peripheral blood mononuclear cells (PBMC) including CTCs are enriched and then placed on the slides. After fixed by 10% formaldehyde solution, cells are hybridized with specifically designed target probes. The fluorescent signal is amplified by a series of nucleic acid hybridization and imaged by fluorescent microscopy for CTCscope analysis. The schematic of CTCscope assay procedure was showed in Figure 1. Compared to the current CTCs detection methods, CTCscope approach is more sensitive and allows single molecule detection in situ in individual cells by using a novel system of probe design and signal amplification. This ability makes CTCscope a powerful platform in routine clinical assays for CTCs detection.

### Tumorgenesis research

*In vivo* study, Burd et al. used standard homologous recombination procedures to target firefly luciferase on the SV40 polyadenylation site to exon 1α of the endogenous p16^INK4a^ gene [12]. Then they applied RNA in situ hybridization (ISH) by using RNAscope 2.0 technology to detect this faithfully reports expression gene, which serves as a tumor suppressor and aging biomarker. Their work suggests that p16^INK4a^ activation is a characteristic of all emerging cancers and it could be set as a sensitive, unbiased reporter of neoplastic transformation. While *in situ* RNA study, Staudt et al. applied macrophage inflammatory protein-1a/CCL3-specific RNA target probe set, which targeted nucleotides 23 to 771 of the CCL cDNA sequence to detect CCL3 mRNA in tumor samples [13]. CCL3 is a chemokine, which is known to amplify inflammation. They found inhibiting NF-ҡB reversed the increase in CCL3 expression associated with LDL receptor-related protein (LRP1) gene silencing in macrophage-like cells. LRP1 is a type 1 transmembrane receptor, which mediates ligand endocytosis and impact cell migration. This phenomenon suggested to prevent LRP1 down regulation in myeloid cells might suppress monocyte recruitment to tumors and cancer angiogenesis.

### EMT biomarkers development

Yu et al. optimized microfluidic capture of CTCs with epithelial- and tumor-specific antibodies, and they then applied this technology to analyze epithelial-mesenchymal transition (EMT) in CTCs from breast cancer patients [11]. They established quantitative, dual-colorimetric RNA-ISH assay to examine the expression of seven pooled epithelial (E) transcripts and three mesenchymal (M) transcripts in tumor cells. In their research, they found that all three major histological subtypes of invasive breast cancer (i.e. ER+/PR+ subtype; HER2+ subtype; and the ER–/PR–/HER2– triple negative subtype) contained rare tumor cells with epithelial morphology that stained with both E and M markers, while that benign breast tissue and tumor cells in pre-invasive ductal carcinoma in situ (DCIS) lesions and reactive stromal cells were exclusively epithelial or mesenchymal. By comparing CTC features in pre- and post treatment blood samples from clinical cases, they found patients who responded to therapy showed a decrease in CTC numbers and/or a proportional decrease in M + compared with E+ CTCs after treatment. In contrast, the patients who had progressive disease while on therapy showed an increased number of M+ CTCs in the post treatment samples.

### Cancer diagnostic research

Payne et al. applied a probe set against mRNAs encoding cytokeratin 8, 14, 17, 18, 19 and 20, EpCAM, and MUC-1 (traditional epithelial cell markers) to detect epithelial cells and another probe set with different fluorescent color was used to detect three genes expressed in tumor cells that have undergone EMT (Twist, N-Cadherin, and fibronectin) expressed in CTCs which have undergone EMT [4]. They found that breast cancer cells could be identified by strong pan-CK staining, whereas the surrounding PBMCs showed minimal fluorescent signals (Figure 1D). Also they found different EGFR mRNA expression levels consistent with the known EGFR protein expression status. The advance of application of fluorescence *in situ* hybridization technique is to detect the gene translocation. Tana et al. applied RNAscope formalin fixed paraffin embed (FFPE) assay in epithelioid hemangioendothelioma (EHE) tissue sections to detect the translocation (the chimeric WWTR1/CAMTA1 transcription factor), which would assist in the evaluation of this diagnostically challenging neoplasm as well as may represent a therapeutic target for EHE [14]. By multispectral imaging system, WWTR1/CAMTA1 fusion transcripts presented yellow punctate dots, while wild-type WWTR1 and CAMTA1 transcripts were stained with red or green punctate dots, respectively. Among these studies, it confirms that CTCscope provides additional prognostic and predictive information in therapy monitoring.

### Cancer stem cell research

Currently, *In situ* study, by RNAscope technology, groups of cancer stem cell biomarker was confirmed. Barry et al. applied RNAscope for Olfm4, which marks crypt base columnar (CBCs) stem cells in intestinal tissue, and to determine if intestinal stem cell (ISC) numbers was reduced by YAP (a protein known as its powerful growth-inducing and oncogenic properties) expression [15]. And in their research, they found epithelial-specific expression of YAP suppressed intestinal cell renewal, which occurred through inhibition of the Wnt-singaling pathway. Ziskin et al. also applied multiplex fluorescent ISH and chromogenic non-isotopic ISH in 57 colorectal adenocarcinomas to detect 19 putative intestinal stem cell markers [16]. They found the G protein-coupled receptor (lgr5) and intestinal stem cells signature gene (Ascl2) was expressed on mainly colorectal cancers, which supported the hypothesis that the cancer cells were derived from lgr5^+^/Ascl2^+^ crypt stem cells. Yan et al. applied fluorescence in situ hybridization (FISH) to detect lgr5 mRNA and genetic signature of pan- yellow fluorescent protein (YFP) expression within the Polycomb group protein (Bmi1) + clonally derived spheroids [17]. The result indicated that the Bmi1+ ISC lineage could generate Lgr5+ cells *in vitro*.

### Other application

On several virus infection models, CTCscope became a popular research tool for detection certain mRNA expression. In order to confirm Hamster-adapted Sin Nombre Virus (HA-SNV) replication in pulmonary endothelial cells, Safronetz et al. applied monoclonal antibodies targeting the nucleoprotein (anti-SNV NP clone 5 F1/F7) nucleotides) and polyclonal rabbit anti-CD31 antibodies in a HA-SNV infection model [18]. They found that virus antigen was predominantly expressed in CD31-positive cells in lung tissues obtained from virus infected hamsters. Another virus research group, Ouwendijk et al. analyzed consecutive ganglionic sections in a reactivated Simian Varicella Virus (SVV) model, by ISH for SVV open reading frame 61 (ORF61) antisense RNA and IFNγ-inducible protein-10 (IP-10/CXCL10) transcripts [19]. The abundance of T cell clusters were correlated with CXCL10 RNA, but not with those of SVV ORF61 antisense RNA. So they concluded after SVV reactivation and transient T-cell infiltration, possibly medicated by CXCL10. Recently, Bishop et al. reported the development of RNA ISH probes complementary to E6/E7 mRNA, which permits direct visualization of human papillomavirus (HPV) transcripts, in routinely processed tissues and provide an accurate HPV detection method for the clinical physicians. Besides, they found p16 expression was strongly associated with the presence of HPV E6/E7 mRNA [20]. By comparison, they confirmed a high rate of concordance (99%) between the E6/E7 mRNA method and HPV DNA ISH.

## Conclusions

This recently developed new technique, CTCscope, is designed for detection mRNA expression on CTCs. Due to its advantage of highly sensitivity, RNA ISH empower its high sensitivity and specificity to define low expression of commonly used biomarkers. However the future application of CTCscope from bench to bed-side seems both promising and challenged. Certain limitations of this approach may hamper its clinical use, e.g. the assay protocol which contains multiple steps is not easy to perform in a clinical lab, the changing of hybridization probes against the target mRNA may reduce the reliability of the detection, and the cut off value and clinical significance of CTC numbers need to be determined for different cancers.

## Abbreviations

CTCs: Circulating tumor cells
CBCs: Crypt base columnar
DCIS: Ductal carcinoma in situ
E: Epithelial
EMT: Epithelial-mesenchymal transition
EHE: Epithelioid hemangioendothelioma
FISH: Fluorescence in situ hybridization
FFEP: Formalin fixed paraffin embed
HA-SNV: Hamster-adapted Sin Nombre Virus
HPV: Human papillomavirus
ISH: In situ hybridization
ISC: Intestinal stem cell
M: Mesenchymal
ORF61: Open reading frame 61
SVV: Simian Varicella Virus.
